# A survey on helminthic infection in mice (*Mus musculus*) and rats (*Rattus norvegicus *and *Rattus rattus*) in Kermanshah, Iran

**Published:** 2013

**Authors:** Norollah Pakdel, Soraya Naem, Farid Rezaei, Abdol-Ali Chalehchaleh

**Affiliations:** 1 *Department of Pathobiology, Faculty of Veterinary Medicine, Razi University, Kermanshah, Iran; *; 2 *Department of Pathobiology, Faculty of Veterinary Medicine, Urmia University, Urmia, Iran.*

**Keywords:** Helminth, Iran, Kermanshah, Mice, Rats

## Abstract

Parasitic infections of rodents can compromise scientific research as well as the health of the animals and humans. Based on previous studies, infection rate of parasitic helminths is different in various regions of Iran. The current survey was aimed to determine endoparasitic helminths infection in 138 trapped rodents of Kermanshah county, Iran. Mice and rats were trapped using metal snares from January to October 2011 and euthanized. Rodents included 110 *Mus musculus* (79.00%), 23 *Rattus norvegicus* (17.00%), and five *Rattus rattus* (4.00%). The gastrointestinal and respiratory tracts were removed and examined to identify parasitic helminths. The results indicated that 42.02% of examined rodents were infected with eight helminths species, i.e. *Trichuris muris* (14.49%), *Syphacia obvelata* (13.76%), *Syphacia muris* (2.89%),* Aspicularis tetrapetra* (5.07%), *Heterakis spumosa* (5.07%),* Capillaria hepatica* eggs (3.62%), *Hyminolepis diminuta* (12.30%), and *Cystisercus fasciolaris*, the larva of *Taenia teanieformi*s (4.34%). Given the results of this study, we concluded that examined rodents were more infected with nematodes than other helminths. As rodents are usually infected with a number of zoonotic parasites, hence control of these animals has an important role in safeguarding public health.

## Introduction

Rodents are one of the most successful, abundant, and destructive group of animals causing direct and indirect damage to agricultural products both at pre and post-harvest stages. In addition, they are responsible for transmitting various agents, including a number of helminths parasites, to human and domestic animals.^1^^-^^3^ Infection in human generally occurs directly through contact with rodent excrement, ingesting food contaminated with their fur, feet, urine or fecal dropping, rodent’s bites and indirectly through bites from ectoparasitic vectors such as flea and ticks.^2^ Wild rodents serve as reservoir host and have greater ability to harbor a number of endoparasitic agents that play important role in human and livestock health.^2^^,^^4^^-^^6^ Helminths such as *Trichinella*, *Angiostrongylus*,* Capillaria*, *Hymenolepis*, *Railleitina*, *Echinococcus*, *Schisto-soma*, *Paragonimus*, and *Echinostoma* that reported from rodents are importance in public health.^6^^-^^8^ In addition, some of rodents' endoparasites such as *Capillaria hepatica* and *Angiostrongylus cantonensis* cause severe syndromes in humans and other animals.^9^^,^^10^ Thus, investigation on rodents’ parasites in different geographical areas has medical and veterinary importance to prevent transmission of diseases to humans and animals.^11^ Several studies have been conducted on parasites of wild rodents from different part of the world that reveal the occurrence of a rich parasite diversity including the endoparasitic helminths fauna^5^^,^^12^^-^^14^ and ectoparasitic arthropods fauna, as well.^15^ In Iran, there are some reports on the occurrence of parasitic infection in different species of rodents in some areas.^16^^-^^20^ In addition, it is demonstrated that some rodents’ species are reservoir of cutaneous leishmaniasis^21^^-^^23^ and visceral leishmaniasis^24^ However, little is known about helminths’ infection in some areas of Iran such as Kermanshah. The present study reports, for the first, the prevalence of mice and rats parasitic helminths in this region.

## Materials and Methods

**Study area. **Rodents for this survey obtained from both urban and rural area of Kermanshah county, southwest Iran (34°18′N, 47°3′E and 1420 m above sea level), and then examined for parasitic helminthic infection. Kermanshah is situated between two cold and warm regions and enjoys a moderate and mountainous climate. It rains most in winter and is moderately warm in summer. The annual rainfall is 500 mm. The average temperature in the hottest months is above 22 ˚C.^25^

**Animals. **A total number of 138 rodents belonging to three species were collected from 56 locations of Kermanshah county from January to October 2011. These animals were trapped using metal snares, and different baits such as fresh cucumber, cheese and walnut. Traps were set at outdoors in agricultural, horticultural and animal farms, dry riverbeds, parks and other suitable places in both urban and rural areas. Trapped rodents were transferred to Parasitology Laboratory, School of Veterinary Medicine, Razi University, Kermanshah, and then euthanized. Each rodent sex was recorded, and identification of the species was confirmed on the basis of morphological characteristics with reference to keys^26^ in Zoology Department of Razi University. After dissection, internal organs (esophagus, stomach, small and large intestines, liver, lungs, peritoneum, urinary bladder, pectoral and abdominal cavity) of each rodent’s carcass were removed and examined for adult or larval stages of helminths under stereomicroscope. Parasites were removed carefully from infected organs, cleared, stained, and identified by using appropriate systematic keys.^27^ In addition, some tissue smears prepared for screening of *Capillaria hepatica* eggs from infected livers. 

## Results

During the course of the study, out of 138 captured animals, 110 were *Mus* *musculus* (43 female; 67 male), 23 were *Rattus norvegicus *(10 female; 13 male), and 5 were *Rattus rattus *(1 female; 4 male) (Table 1). Forty two percent (58/138) of examined animals were infected with different helminths whose 21.10% (30/73) trapped in urban areas and others, 20.90%, (28/65) captured in rural areas. 

**Table 1:** Distribution of trapped rodents based on sex and location (F: Female, M: Male).

**Location of sampling**	***Mus musculus***		***Rattus norvegicus***		***Rattus rattus***		**Total**
	**M**	**F**	**M**	**F**	**M**	**F**	
**Urban area**	22	31	7	10	1	2	73
**Rural area**	21	36	3	3	0	2	65
**Total**	43	67	10	13	1	4	138

**Fig. 1:**
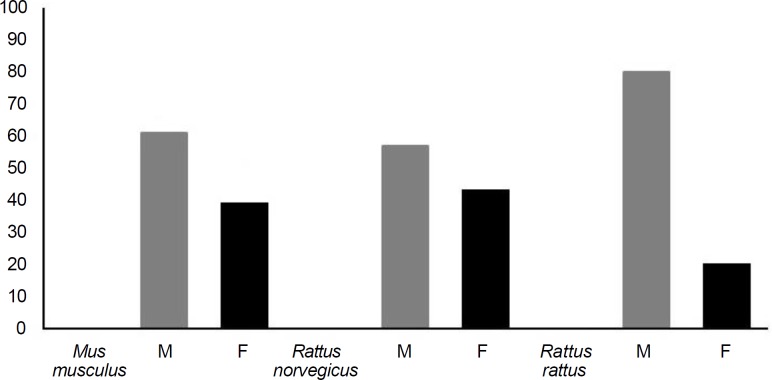
Infection rate (%) based on species and sex (χ^2 ^= 137.087, df = 2), (F: Female, M: Male).

**Fig. 2:**
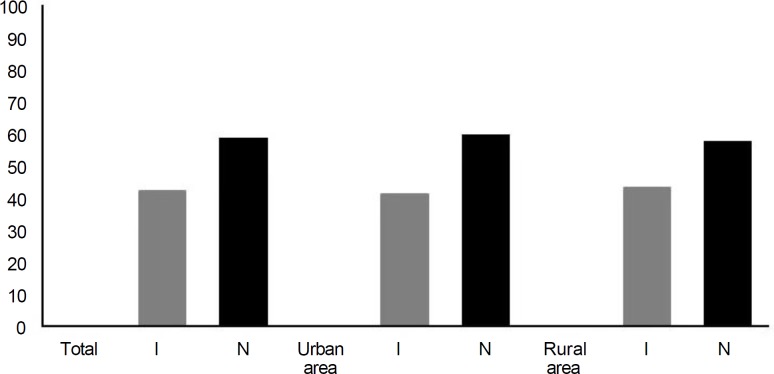
Distribution rate (%) of infected (I) and non-infected (N) mice based on locations of sampling (χ^2 ^= 0.069, df = 1).

Figure 1 shows infection rate based on mice species and sex and Fig. 2 demonstrates the distribution of infected (I) and none infected (N) rodents based on geographical distribution. Five species of nematodes (*Syphacia obvelata*, *Syphacia muris*, *Aspicularis tetrapetra*, *Heterakis spumosa* and *Trichuris muris*), one species of adult cestodes (*Hymenolepis diminuta*) and one species of larvae form of cestodes (*cysticercus fasciolaris*) were identified. In addition, the eggs of *Capillaria hepatica* were identified from 3.62% of examined rodents. Of 73 trapped rodents in urban areas, 23 (32.00%) were infected with nematodes, 14 (19.00%) with cestodes, and 7 (9.50%) showed mixed infection. These results in rural areas were; 23 rodents (35.00%), 9 rodents (14.00%), and 4 rodents (6.00%), respectively. Also, a total number of 58 infected rodents, 36 (62.06%) were female, and 22 (37.94%) were male. The results indicated that examined rodents were more infected with nematodes than cestodes (*p *≤ 0.05, χ^2 ^= 23.725, df = 2).* Trichuris muris* had the highest prevalence and *Syphacia muris* the least abundant. Infection rates in those mice involved with *Aspicularis tetrapetra* (5.07%)*, Heterakis spumosa, (5.07%) *and* Cysticercus fasciolaris *(4.34%), were nearly similar. 

**Fig 3:**
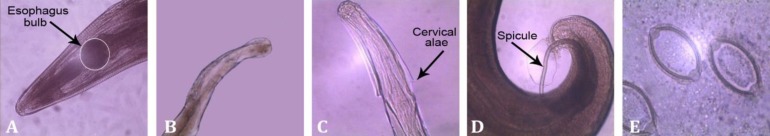
**A) **Anterior end and esophagus bulb of *Syphacia obvelata*, 400 ×; **B)** Anterior end and cervical alae of *Syphacia muris*, 100×; **C)** Anterior end of *Aspicularis tetrapetra*, 400 ×; **D)** Posterior end of *Trichuris muris* (male), 400 ×; **E)** *Capillaria hepatica* eggs in the liver of infected rodents, 400 ×.

In Table 2, infection rates of examined rodents with different helminths based on animals’ species are shown. From 84 identified *S. obvelata*, 12 helminths (14.00%) were male and 72 helminths (86.00%) were female. Also, 70.00% (7 helminths) of *S. Muris* was female, and 30.00% (3 helminths) was male. The ratios for *A. tetrapetra*, *H. spimusa* and *T. muris* were 27.00% (12 worms) male and 73.00% (33 helminths) female, 54.00% (30 worms) male and 46.00% (26 helminths) female, and 33.00% (34 helminths) male and 67.00% (70 helminths) female, respectively. Figure 3 shows some of parasitic helminths removed from examined mice. No infection was observed in the esophagus, stomach, and lung of examined animals.

**Table 2:** Infection rates (%) of identified helminths in examined mice.

**Mixed ** **infections **	***Cystisercus fasciolaris ***	***Hyminolepis diminuta ***	***Capillaria hepatica*** ** eggs **	***Trichuris muris ***	***Heterakis spumosa ***	***Aspicularis tetrapetra ***	***Syphacia muris ***	***Syphacia obvelata***	**Specie of rodent**
10.90	4.54	9.09	0.90	16.36	3.64	6.36	4.54	14.54	***Mus musculus***
8.70	3.34	30.43	13.04	8.69	13.04	0	0	4.34	***Rattus norvegicus***
0	0	0	20.0	0	0	0	0	20.00	***Rattus rattus***

## Discussion

The present study gives the first overview on the endoparastic helminths infection of trapped rodents in Kermanshah, Iran of which reporting eight species of helminths from three species of rodents. Data from previous studies on helminths parasites in rodents of other regions of Iran were partially comparable with the helminths fauna in the present study. In this study, the number of trapped *Mus musculus* was significantly higher than other species of rodents (χ^2 ^= 137.087, df = 2,* p *≤ 0.05), but no significant differences between infection rate in urban and rural areas was observed (χ^2 ^= 0.069, df = 1). Also, infection with nematodes was significantly higher than cestodes (χ^2 ^= 23.725, df = 2, *p *≤ 0.05), while the trematodes were absent in examined mice, resembling the results of another report by Malsawmtluangi and Tandon in India.^6^

Infection rate in male animals (62.06%) was higher than female (37.94%), this in irreconcilable with finding of Milazzo *et al*. who found no significant differences between male and female rodents.^8^ However Rogriguez *et al*. found higher prevalence of helminths in male rodents.^28^ Of identified nematodes from infected rodents, the number of female helminths (208 helminths) was higher than male helminths (91 helminths) (χ^2 ^= 45.873, df = 1, *p *≤ 0.05). *Trichuris muris* was the most common nematode removed from infected animals, but no significant differences were found among this parasite and *S.* *obvelata* and *H. diminuta*, which were nearly similar to those findings of other reports in Iran^11^^,^^29^^-^^32^ and other locations of the world.^8^^,^^33^^-^^35^ *Hymenolepis nana*,^36^ *Syphacia hodarae* *n. sp*.,^37^ *Trichuris navonae* *n. sp*.,^38^ *Gongylonema monigi,*^19^ *Physaloptera* *spp*.,^32^^,^^39^ *Nipostrongylus brasiliense,*^40^ *Angiostrongylus cantonensis,*^41^^,^^42^ *Strongylus ratti,*^27^ and larvae of *Taenia endorasicus*^43^ were reported in some previous investigations, but were absent in current study. In addition, Asmar *et al*. and Kia *et al*. reported *Strongyloides ratti* and *Physaloptera sexulatus* whom were not observed in this study.^19^^,^^44^ Singla *et al*. indicated that *C. fasciolaris* were the common helminths in rodents of Panjab, India, and its prevalence was much higher (35.20%) than our finding (4.34%).^2^ In other investigations, infestation of rodents with ectoparasites^35^^,^^45^ and protozoa (e.g. *Cryptosporidium spp*. and *Sarcocystis spp*.)^30^^,^^39^ have also been reported.

Some of the recovered parasites from rodents in this study were of zoonotic importance helminths, including *C. hepatica*, *H. diminuta*, *S. obvelata*, and *Taenia taeniaformis* larva. *Hymenolepis nana *is the zoonotic helminth commonly reported in Iran,^46^ but it was not found in this study. Also *H. diminuta* has already been reported in human.^47^^,^^48^ This parasites are common in children and sometimes produce disorders in the hosts.^49^ *Capillaria hepatica*, which was found in this survey, is very important in human causing a lethal syndrome which has already been reported from different countries.^10^ These zoonotic parasites have been reported from rodents in variable prevalence of different areas worldwide such as Siberia,^9^ Switzerland^50^ and Iran.^18^ Because of important role of rodents in spreading different parasitic agents and destroying the food crops, control programs are needed for reducing their adverse impact.
